# Bibliography

**Published:** 1897-04

**Authors:** 


### Bibliography.

A Practical Treatise on Artificial Crown- and Bridge-Work. By George Evans, Lecturer on Crown- and Bridge-Work at the Baltimore College of Dental Surgery, etc. Fifth edition, revised. With six hundred and twenty-five illustrations. The S. S. White Dental Manufacturing Co., Philadelphia, 1896.

The issuing of a fifth edition of a work in a few years is a sure evidence that it has been appreciated by those it has been intended to serve. This work of Dr. Evans has passed the stage of criticism, and has become a standard text-book upon the subjects treated.

It was reviewed in this journal in its third edition in 1893, and since then it has steadily improved. "Obsolete matters have been omitted, descriptions of unimportant variations have been curtailed, repetitions avoided as far as possible."

The explanations of the various processes in forming crowns are clear and concise, and it would seem as though any one familiar with plate-work could not fail to become skilled in the work by faithfully following these in practice. Unfortunately this is not always the case, and unskilful work in preparation and insertion is too frequently in evidence.

A considerable portion of the book is devoted to "bridge-work," permanent and removable. It is a question whether permanent bridge-work will exist as part of dentistry in the coming time. There is a growing feeling, in which the writer sympathizes, that this process is fast reaching the stage of a professional abomination. The fact that so much space is given to removable bridge-work indicates the drift of sentiment away from earlier methods. That the author does not quite agree with this view is to be expected, and he, therefore, devotes time and space to its elucidation.

As a text-book for students and reference-book for advanced practitioners this work of Dr. Evans can most cordially be recommended, and it is probable it will remain without a competitor in this line of work for a long period.

